# Clinical validation of the normalized mutual information method for registration of CT and MR images in radiotherapy of brain tumors

**DOI:** 10.1120/jacmp.v5i3.1959

**Published:** 2004-10-21

**Authors:** Theo Veninga, Henkjan Huisman, Richard W. M. van der Maazen, Henk Huizenga

**Affiliations:** ^1^ Department of Radiation Oncology University Medical Center Nijmegen P.O. Box 9101, 6500 HB Nijmegen the Netherlands; ^2^ Department of Radiology University Medical Center Nijmegen P.O. Box 9101, 6500 HB Nijmegen The Netherlands

**Keywords:** image registration, normalized mutual information, registration accuracy, validation protocol, matching CT‐MR

## Abstract

Image registration integrates information from different imaging modalities and has the potential to improve determination of target volume in radiotherapy planning. This paper describes the implementation and validation of a 3D fully automated registration procedure in the process of radiotherapy treatment planning of brain tumors. Fifteen patients with various brain tumors received computed tomography (CT) and magnetic resonance (MR) brain imaging before the start of radiotherapy. First, the normalized mutual information (NMI) method was used for image registration. Registration accuracy was estimated by performing statistical analysis of coordinate differences between CT and MR anatomical landmarks along the *x‐, y‐* and *z‐*axes. Second, a visual validation protocol was developed to validate the quality of individual registration solutions, and this protocol was tested in a series of 36 CT‐MR registration procedures with intentionally applied registration errors. The mean coordinate differences between CT and MR landmarks along the *x‐* and *y‐*axes were in general within 0.5 mm. The mean coordinate differences along the *z‐*axis were within 1.0 mm, which is of the same magnitude as the applied slice thickness in scanning. In addition, the detection of intentionally applied registration errors by employment of a standardized visual validation protocol resulted in low false‐negative and low false‐positive rates. Application of the NMI method for the brain results in excellent automatic registration accuracy, and the method has been incorporated into the daily routine at our institution. A standardized validation protocol ensures the quality of individual registrations by detecting registration errors with high sensitivity and specificity. This protocol is proposed for the validation of other linear registration methods.

PACS numbers: 87.53.Xd, 87.57.Gg

## I. INTRODUCTION

Correct determination of tumor localization and extension is of major importance in radiation oncology. This is especially true from the perspective of modern radiation treatment techniques such as inverse planning and intensity‐modulated radiotherapy. These techniques offer the possibility of dose escalation and improved sparing of normal tissues. The precise delineation of gross tumor volume is one of the quality assurance aspects that have to be dealt with when applying these techniques. Various imaging techniques such as computed tomography (CT) and magnetic resonance (MR) imaging each provide specific and essential information for tumor staging. A correct interpretation of this information requires an accurate image registration. This can be achieved by attaching a stereotactic frame or a set of fiducial markers to the skull before performing all imaging procedures. For radiotherapy purposes, it is more convenient to employ a retrospective registration procedure to incorporate the available image volumes from different imaging modalities into one correlated data set.

Some of these registration methods are highly interactive and require the localization of corresponding landmarks^(^ ^1^ ^–^ ^3^ ^)^ in different data sets. Chamfer or surface matching^(^ ^4^ ^–^ ^6^ ^)^ employs an automated algorithm to align a set of contours in one data set to a generated point‐distribution (depicting the same region of interest) in a second data set. An alternative approach is to calculate the transformation from one data set to another by correlating the intensity values of corresponding voxels.^(^ ^7^ ^–^ ^12^ ^)^ One such mathematical procedure is called mutual information and is proposed to optimize the correlation of these intensities and calculate the desired 3D transformation for image registration.^(^ ^13^ ^,^ ^14^ ^)^ Studholme et al.^(^ ^15^ ^,^ ^16^ ^)^ demonstrated the utilization of this method in a clinical setting.

In a comprehensive collaborative study, West et al.^(17)^ evaluated a number of registration techniques. Although providing generally satisfactory results, some techniques resulted in large maximum errors. Currently, no registration method performs optimally (i.e., within certain error margins) for every patient. Incorrect registration of image data and, hence, incorrect target volume delineation result in an incorrect radiotherapy plan. Since this is unacceptable, the clinical implementation of any registration procedure should be accompanied by a validation procedure. Surprisingly, despite the rapidly growing employment of commercially available “fusion” software in radiotherapy centers, clinical validation procedures for registration are scarcely covered in the literature.

In this paper, we describe our experience with the validation of the clinical implementation of the normalized mutual information (NMI) method. Registration of CT and MR was carried out in a consecutive series of 15 patients who were scheduled for cranial radiotherapy. First, registration accuracy was estimated by performing statistical analysis of coordinate differences between CT and MR anatomical landmarks. Second, a visual validation protocol was developed to validate the quality of individual registration solutions. The objective of this protocol is to reject the registration solution in the case of registration errors that exceed a certain threshold value. The sensitivity and specificity of the validation protocol were tested in a series of misregistrations with intentional registration errors similar to what might occur in clinical practice.

## II. METHODS

### A. Patient selection

For this study, we used the image data sets of 15 patients who were referred for cranial irradiation. These patients were diagnosed with a primary brain tumor such as an astrocytoma or oligodendroglioma or with a solitary brain metastasis from an extracranial tumor. All patients had both “planning CT” and MR scans of the brain for the purpose of radiotherapy treatment planning.

### B. Data acquisition

CT imaging was performed with a Siemens Somatom Volume Zoom scanner after IV administration of contrast fluid (Omnipaque) (140 kVp, 146 mA, axial acquisition, 512×512 pixels, pixel size 0.5×0.5×2 mm , standard head reconstruction kernel). The reconstructed slice thickness (RST) was 3 mm for nine patients. Two patients had RST of 2 mm, and four patients had RST of 5 mm. MR examination consisted of a 3D T1‐weighted magnetization prepared rapid gradient echo (MP‐RAGE) sequence (TR 11.4 ms, TE 4.4 ms, #ave=1, matrix size 256×256×123 mm, RECT FOV 256 mm, voxel size 1×1×2 mm, slice thickness 2 mm, slice gap=0 (3D), bandwidth 60 Hz/pixel, use of head coil). The MR data of four patients were obtained with a 2D spin‐echo (SE) technique (voxel size 1×1×5 mm). Data acquisition was performed at 1.0 T (Magneton 63/84 SP/4000, Siemens Medical, Erlangen, Germany).

### C. Registration procedure

Registration of the resulting 3D CT and MR data sets was conducted with the NMI method as described by Studholme et al.^(^ ^15^ ^,^ ^16^ ^)^ The software package “mpr” (written by Studholme and available at http://noodle.med.yale.edu/~cs/software/) was embedded in an in‐house developed user interface using Tcl/Tk scripting. The OFFIS dcmtk was also embedded to provide DICOM compatibility. The software ran under Solaris on a Sun SparcStation‐20, and registration took 30 min of calculation time. The image registration method first isotropic resampled the input images using tri‐cubic interpolation. Resampling took place at three different resolutions to form multi‐resolution images. Isotropic images were aligned by maximizing the NMI. Normalized mutual information was estimated using all overlapping image voxels with a discrete joint histogram of 64×64 bins. Linear interpolation was used during optimization. Initial registration was obtained at the lowest resolution (6 mm) and gradually refined until the highest resolution (1.5 mm).

### D. Estimation of registration accuracy

Residual registration errors after registration were estimated by measuring the coordinate differences along the *x‐* (“left‐right”), *y‐* (“anterior‐posterior”), and *z‐*axes (“cranio‐caudal”) between a set of well‐defined landmarks on CT and MR. The lateral, anterior, and posterior boundaries of the skull are well recognized on CT and MR and were used as landmarks for estimating *x‐* and *y‐*coordinate differences. Measurements were carried out at six transversal levels with 1.5‐cm spacing. The means of these differences were used as an estimate for registration accuracy along the *x‐* and *y‐*axes. Coordinate differences along the *z‐*axis were measured by determining the *z‐*coordinate of four anatomical landmarks: roof of skull bone (RSB), bottom of sella turcica (BST), cranial border of mastoid bone (CBM), and top of dens axis (TDA). The mean of these differences served as an estimate for registration accuracy along the *z‐*axis. In addition, the centers of the right and left eye globe were used as an estimate for registration errors in 1D (along *x‐, y‐* and *z‐*axes) as well as in the 3D direction by calculating the vector length for the combined *xyz‐*coordinate difference.

All measurements were carried out on a CadPlan^(18)^ workstation to which the image data were transferred using a dedicated, homemade utility. We recognize that the accuracy of the determination of coordinate differences between CT and MR landmarks after applying the automated NMI registration is limited by the resolution of the image data sets as well as the accuracy with which well‐defined anatomical landmarks can be aligned manually (i.e., by eye).

### E. Independent visual validation protocol

**Figure 1:**
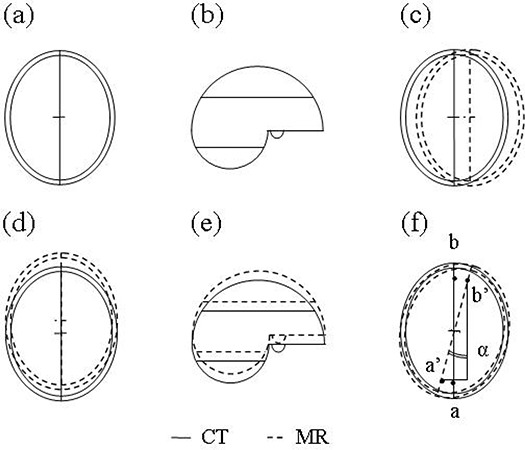
Correct (a), (b) and examples of incorrect registration of CT and MR data sets with translation errors along *x‐*axis (c), *y‐*axis (d), or *z‐*axis (e). As a result of *mis*registration with rotation error (angle α) around the *z‐*axis, the points a and b are depicted at positions a’ and b’ (f).

For the purpose of individual quality assurance (QA) of image registration, an independent visual validation protocol was developed, using “3D Slicer,” a freely available software program for visualization and processing of medical data.^(19)^ All image data were transferred to 3D Slicer in DICOM format. The validation protocol consists of a stepwise procedure for an estimate of translation errors along the three orthogonal axes $({\text{Tr}}_{x,}{\text{Tr}}_{y,}$ and ${\text{Tr}}_{z})$ as well as rotation errors around these three axes $(R_{x},R_{y}$, and $R_{z})$ (Fig. 1).

The procedure is as follows:

Validation of translation

(a) *xy‐*axis

Possible translation errors were estimated at three consecutive *z‐*levels just above the mastoid bone with a spacing of 10 mm. The transition from brain parenchyma to CSF space and the anterior and posterior attachments of the falx cerebri were used as reference points.

(b) *z‐*axis

Detection of possible *z‐*errors was carried out by viewing the data sets in coronal and sagittal orientation at the “midplane” level. In addition to the four landmarks as described before (RSB, BST, CBM, and TDA), it was optional to use other landmarks (e.g., tentorium cerebelli, bony palatum, and nasal septum) to quantify *z‐*errors.

Validation of rotation

(c) *x‐*axis

The data sets were viewed in three sections with sagittal orientation, that is, at midplane and to the left and the right with 3‐cm spacing. The lateral sections “cut” the eyeballs in half in order to use them as reference points. Additional landmarks included the os nasale, the bony palatum, the posterior attachment of the tentorium cerebelli, and the sulci of the brain parenchyma.

(d) *y‐*axis

Two coronal reconstructions were made at the anterior and posterior part of the corpus callosum. The third section was taken about halfway along this structure in order to include the pituitary gland and sphenoid bone as reference points. The cranial and lateral attachments of, respectively, the falx cerebri and tentorium cerebelli as well as the brain sulci served as additional landmarks for orientation.

(e) *z‐*axis

Three axial slices were used that were identical to those used for detection of *xy* translation errors. Two landmarks were crucial with respect to these kinds of errors: the anterior and posterior attachments of the falx cerebri.

### F. Detection of intentionally applied misregistrations using the validation protocol

A test procedure was designed to confirm the ability of the validation protocol (see previous paragraph) to detect the presence of deliberately introduced registration errors. For this purpose, the original registration outcomes of two patients were selected that were judged as being optimal, based on visual assessment of registration accuracy. Each registration outcome consisted of a CT and an MR data set. The CT data set had a reconstructed slice thickness of 3 mm, while the MR data set was acquired with a 3D MP‐RAGE sequence with voxel size 1×1×2 mm (further specifications, see Data acquisition). The original registration outcomes were manipulated by randomly introducing translation or rotation errors in the range 0 mm to 5 mm (translations) and 0° to 5° (rotations). This was achieved by changing the translation and rotation parameters of the registered CT data sets. This resulted in a series of 36 simulated misregistrations that contained a total of 41 intentionally applied registration errors. These consisted of 24 translation errors and 17 rotation errors, thereby representing all error categories. The majority of registrations (28) contained one registration error, five registrations contained two errors, and one registration contained three registration errors. Two registrations contained no registration errors. A practiced radiation oncologist (T.V., who was blinded to the result of the intentionally applied registration errors) used the validation protocol (incorporated in 3D Slicer) to judge the presence or absence of any registration errors in these series as well as to estimate the magnitude of errors if any. Since every registration potentially contains six registration errors (three translation and three rotation errors), this resulted in a total of 6×36=216 observations in 36 data sets.

These observations were used to calculate sensitivity and specificity as well as false‐positive (FP) and false‐negative (FN) rates. For this purpose, we defined error thresholds that serve as an upper limit of registration errors that would still be accepted. The hypothesis was that the validation protocol was capable of detecting all errors that exceeded these thresholds. For translation errors we accepted an error threshold of 1 mm, which is within 2 standard deviations (SD) of the registration accuracy as estimated with CadPlan (Table 1). For rotation errors a maximum misregistration angle of 1° was accepted. The choice of the thresholds reflects the accuracy of the manual alignment of landmarks, imperfections of the image data sets (including voxel size, reconstructed slice thickness, and partial volume effects), as well as the desired accuracy for radiotherapy treatment planning.

**Table 1:** Coordinate differences (mm) between CT and MR along the x‐, y‐ and z‐axes after registration of data sets with RST of 2 mm to 3 mm (CT) and MR voxel size of 1×1×2 mm ^a^ ^,^ ^b^

Registration	RST	*x*		*y*		*z*	
		mean	max	mean	max	mean	max
1	2	0.1	1.0	0.5	1.5	0.5	2.0
2	2	0.1	1.0	0.1	1.5	0.0	0.0
3	3	0.2	1.0	0.7	2.0	0.5	2.0
4	3	0.1	1.0	0.3	2.5	0.0	0.0
5	3	0.3	1.0	0.1	0.5	0.0	2.0
6	3	0.0	0.5	0.4	1.5	0.0	2.0
7	3	0.1	0.5	0.6	1.5	1.0	2.0
8	3	0.3	1.0	0.8	2.0	0.5	2.0
9	3	0.2	1.5	0.4	1.5	0.5	2.0
10	3	0.3	1.0	0.3	2.0	0.8	3.0
11	3	0.1	0.5	0.1	2.0	0.0	0.0
							
σ		0.5		0.9		1.0	

a MR examination consisted of a 3D magnetization prepared‐rapid gradient echo (MP‐RAGE) sequence (TR 11.4 ms, TE 4.4 ms, voxel size 1×1×2 mm).

b RST =reconstructed slice thickness of CT data set; σ=standard deviation.

## III. RESULTS

### A. Registration accuracy

Eleven CT data sets had a reconstructed slice thickness (RST) of 2 mm to 3 mm and MR voxel size of 1×1×2 mm. The mean coordinate differences between CT and MR anatomical landmarks along the *x‐* and *y‐*axes, as estimated within CadPlan, were within 1.0 mm for all registrations in this series (Table 1). The maximum CT‐MR difference was 2.5 mm along the *y‐*axis for the fourth registration. The SD for the *x‐* and *y‐*axes were 0.5 mm and 0.9 mm, respectively. The mean observed coordinate differences along the *z‐*axis were also within 1.0 mm, with a maximum of 3.0 mm for the tenth registration (SD 1.0 mm). A second series of four patients had an RST of 5 mm and MR voxel size of 1×1×5 mm (Table 2). The mean coordinate differences and SD for these patients were increased with a factor 2 to 3.

**Table 2:** Coordinate differences (mm) between CT and MR along the x‐, y‐ and z‐axes after registration of data sets with RST of 5 mm (CT) and MR voxel size of 1×1×5 mm ^a^ ^,^ ^b^

Registration	*x*		*y*		*z*	
	mean	max	mean	max	mean	max
1	0.7	1.5	1.3	3.0	1.7	5.0
2	0.1	0.5	1.3	2.0	0.0	0.0
3	0.0	0.5	0.6	2.0	2.5	5.0
4	0.3	1.0	0.1	0.5	0.0	0.0
						
σ	0.5		1.2		2.1	

a MR examination consisted of a 2D spin‐echo (SE) multislice technique (voxel size 1×1×5 mm).

b RST =reconstructed slice thickness of CT data set; σ=standard deviation.

Using the center of right (OD) and left (OS) eye globe as reference structure for registration errors gave similar results (Table 3). The mean 3D coordinate differences for the first series of registrations (RST 2 mm to 3 mm, voxel size 2 mm) were 1.4 mm and 1.3 mm for OD and OS, respectively. These values increased to 2.5 mm for both OD and OS for the second series of registrations with larger RST (5 mm) and MR voxel size (1×1×5 mm).

**Table 3:** Coordinate differences (mm) between CT and MR after registration as determined for center of right eye (OD) and left eye (OS) in 1D and as 3D vector length. Registration was performed with a CT data set with an RST of 2 mm to 3 mm and 3D MR with voxel size 1×1×2 mm (11 patients) or with CT data sets with an RST of 5 mm and 2D multislice MR with voxel size 1×1×5 mm (4 patients)^a^

	Axis	RST =2 mm to 3 mm, 3D MR (1×1×2 mm)			RST = 5 mm, 2D MR (1×1×5 mm)		
		mean	σ	max	mean	σ	max
OD							
1D	*x*	0.1	0.4	1.0	0.4	0.6	1.0
	*y*	0.1	0.9	2.0	0.6	0.4	1.0
	*z*	0.8	1.4	4.0	1.9	2.1	5.0
							
3D	*xyz*	1.4	1.3	4.5	2.5	1.6	5.1
OS							
1D	*x*	0.0	0.6	1.0	0.8	0.6	1.5
	*y*	0.4	0.5	1.0	0.3	0.3	0.5
	*z*	0.7	0.9	3.0	1.9	2.1	5.0
							
3D	*xyz*	1.3	0.7	3.1	2.5	1.6	5.0

a RST =reconstructed slice thickness of CT data set; σ=standard deviation.

### B. Independent visual validation protocol

The visual validation protocol was developed to provide a time‐effective and adequate tool to assess the quality of an individual registration solution. After a short “learning period” to become familiar with the stepwise procedure, an inexperienced observer was able to perform this task within 5 to 10 min.

### C. Detection of intentionally applied misregistrations using the validation protocol

The employment of the in‐house developed visual validation protocol resulted in detection of all (24/24) intentionally applied translation errors (Table 4). Similarly, 16 out of 17 rotation errors were detected, whereas one intentionally applied rotation error around the *y‐*axis (magnitude 1°) remained undiscovered. However, in two misregistrations a large difference was observed between the introduced and observed error (6° for *y‐*axis rotation and 4 mm for *z‐*axis translation, respectively). There were 28 false‐positive (FP) measurements (i.e., the detection of a registration error in the absence of such an error), which included 15 FP rotation errors around the *y‐*axis. The majority of FP errors (24/28, 86%) had a size of 1 mm or 1°.

**Table 4:** Detection of intentionally applied registration errors: Sensitivity and specificity rates specified for each error category and for all observations; threshold 1 mm and 1°^a^

		${\text{Tr}}_{x}$	${\text{Tr}}_{y}$	${\text{Tr}}_{z}$	Total (Tr)	$R_{x}$	$R_{y}$	$R_{z}$	Total (R)	Total (Tr+R)
Sensitivity	*n*	7/7	8/8	9/9	24/24	6/6	5/6	5/5	16/17	40/41
	%	0	0	0	100	100	83	100	94	98
Specificity	*n*	23/28	26/28	24/28	73/84	29/30	15/30	30/31	74/91	147/175
	%	82	93	86	87	97	50	97	81	84

a Tr=translation; R=rotation.

Thus, the sensitivity and specificity rates were 100% and 87% for translation errors and 94% and 81% for rotation errors, respectively (Table 4). This resulted in overall (translation and rotation errors combined) sensitivity and specificity rates of 98% and 84%, respectively. The FP rates for translation and rotation errors were 13% (11/84) and 19% (17/91), whereas the FN rates were 0% (0/24) and 6% (1/17), respectively (Table 5).

**Table 5:** Detection of intentionally applied registration errors: False‐positive (FP) and false‐negative (FN) measurements specified for each error category and for all observations.^a^

		${\text{Tr}}_{x}$	${\text{Tr}}_{y}$	${\text{Tr}}_{z}$	Total (Tr)	$R_{x}$	$R_{y}$	$R_{z}$	Total (R)	Total (Tr+R)
FP	*n*	5/28	2/28	4/28	11/84	1/30	15/30	1/31	17/91	28/175
	%	18	7	14	13	3	50	3	19	16
FN	*n*	0/7	0/8	0/9	0/24	0/6	1/6	0/5	1/17	1/41
	%	0	0	0	0	0	17	0	6	2

a Tr=translation; R=rotation.

## IV. DISCUSSION

The spatial information of imaging studies can be integrated into one data set by the employment of a registration procedure. A number of techniques have been described in the literature. A stereotactic fixation frame may be attached to the bony skull during subsequent CT and MR scanning procedures and serve as a reference structure for registration. Although providing a fairly reliable registration method, it requires a controlled clinical setting, and registration can only be carried out prospectively. This disadvantage can be overcome by so‐called retrospective registration methods, which pose less logistical problems and may be more useful for clinical application.

Traditionally, these retrospective methods required the identification or delineation of corresponding structures in subsequent image data sets to perform registration. Pelizzari et al.^(6)^ used a minimum finding algorithm to calculate the coordinate transformation between identical 3D surface models in different data sets (CT, MR, and PET). Van Herk et al.^(20)^ demonstrated the clinical use of chamfer matching by assessing the movement of the prostate during a radiotherapy course after matching the contours of the bony pelvis. Similarly, Maguire et al.^(^ ^3^ ^,^ ^21^ ^)^ utilized the coordinates of anatomical landmarks and/or external markers for correlating CT/MR and PET/SPECT tomographic images. Although generally providing an adequate image registration, these methods suffer from the intensive user interaction that is required. As a consequence, the process is highly user‐dependent and relies on the skill of the user.

An alternative approach is to derive measures from (the spatial distribution of) the respective voxel intensities of each data set, which are then employed as parameters for registration methods.^(^ ^7^ ^–^ ^14^ ^)^ Studholme et al.^(16)^ compared four such similarity measures and concluded that measures of soft tissue correlation^(11)^ and mutual information^(^ ^13^ ^,^ ^14^ ^)^ provided robust solutions for the registration of 3D CT and MR data sets. Freire et al.^(22)^ also demonstrated the robustness of the Geman‐McClure estimator in a comprehensive study of several intensity‐based measures. The results of such automated registration methods are also influenced by the method of interpolation. Tsao^(23)^ showed that the outcome of registration by using mutual information was greatly affected by interpolation artifacts. In addition, registration accuracy depends on several other factors, such as the volume of interest,^(^ ^10^ ^,^ ^16^ ^,^ ^24^ ^)^ the image modalities to be registered,^(24)^ image distortion,^(25)^ patient motion during acquisition,^(16)^ and anatomical changes between scans. Consequently, for the purpose of quality assurance, this paper aimed at the development of a validation protocol for the registration procedure within our institute.

The validation and comparison of registration techniques is impeded by the lack of a “gold standard” for registration. West et al.^(17)^ evaluated 16 registration techniques for CT, MR, and PET images in a collaborative study of 12 centers. The resulting registrations of all centers were compared with a gold standard, which consisted of a prospective registration technique employing fiducial markers. Median errors for CT‐MR and PET‐MR registration were in the range 0.7 mm to 6.3 mm and 2.0 mm to 5.3 mm, respectively. The majority of these techniques resulted in maximum registration errors that exceeded a value of 10 mm with a maximum of 63.2 mm for CT‐MR registration. Pietrzyk et al.^(24)^ validated their interactive registration technique by calculating the SDs of transformation parameters as determined by five experienced users. Fitzpatrick et al.^(26)^ also employed skull‐attached fiducial markers to obtain a gold standard registration solution in a comparative study of three different visual assessment methods of the quality of CT‐MR registration. While no method could reliably detect a registration error of 1 mm, all three methods performed well in detecting errors of 2 mm and higher with agreement rates of more than 75% and mean ROC areas under the curve greater than 0.80.

The NMI method was proposed by Studholme et al., who demonstrated its robustness^(^ ^15^ ^,^ ^16^ ^)^ in a clinical setting. The method was readily available at the time the present research was carried out. These were the primary reasons to choose the NMI method in our study, but we emphasize that alternative registration methods might perform equally. To evaluate its effectiveness in the information environment within our hospital (comprising the specific CT and MR scanning procedures, the method of image data resampling, and method of data transportation), we performed a pilot study on the image data sets of 15 patients. Before discussing these results, it should be recognized that the observed coordinate differences between CT and MR included the inaccuracy of manual landmark determination, which might be inferior to the NMI method.

In the present study, we detected small coordinate differences along the *x‐* and *y‐*axes after registration with the NMI method. The estimated CT‐MR differences and standard errors increased along the *z‐*axis; this can be fully explained by the loss of resolution in cranio‐caudal direction as a result of the shape and size of the MR voxels. A similar increase was observed when MR data‐acquisition (with a 2D sequence) was performed with voxel heights of 5 mm. For these latter data sets, imperfect slice encoding might contribute to the increased CT‐MR differences and standard error along the *z‐*axis. Additional measurements involved both eye globes as reference landmarks and resulted in similar figures. From these results it may be deduced that registration accuracy of the NMI method, as estimated by direct visual assessment of the location of a number of anatomical reference points, was excellent with standard errors close to voxel size. This is in accordance with the results of West et al.^(17)^ and is fully compatible with the required accuracy (1 mm) for radiotherapy treatment planning. A further improvement of registration accuracy can only be achieved by enhancing resolution during the acquisition of image data.

The estimation of registration accuracy, as described above, was performed in CadPlan(18) and has the advantage of integration within the radiation treatment‐planning process. However, it does not allow for easy judgment of rotation errors; in addition, it involves a time‐consuming procedure that in clinical practice hinders an efficient patient flow. Therefore, in addition to estimating registration accuracy in general, we developed a standardized visual validation protocol to “score” the outcome of any individual registration solution in a quick manner. This protocol serves as a quality assurance (QA) measure and, rather than quantifying registration errors exactly, aims at detecting errors that exceed a certain (previously defined) threshold. Since 3D landmarks may not be well defined in all directions, we propose the utilization of a series of 1D defined landmarks in a stepwise procedure. We demonstrated that all intentionally applied registration errors larger than 1 mm (translations) and 1 ° (rotations) were detected, which complies with the required precision (1 mm, 1 °) in modern radiotherapy. Interestingly, only one of nine intentional “small” errors (with values of 1 mm or 1°) remained undetected. Based on the resolution of the data sets (determined by the data acquisition parameters), we had, at least for the small errors, anticipated larger FN rates. The FP rates were obviously higher than the FN rates, especially for detection of rotation errors around the *y‐*axis. This could partially be explained by distortion in the frequency‐encoding direction during MR acquisition. More likely, these rotation errors remained undetected due to the relative lack of reliable anatomic landmarks in the *xz‐*plane (i.e., the coronal plane). Another explanation may be the existence of a (previously undetected) baseline *y‐*axis rotation error in the original registration outcome, which subsequently is recognized by using the validation protocol. This is a direct consequence of the present lack of a gold standard for registration. Nevertheless, the absolute values of the majority (86%) of FP observations did not exceed 1 mm (translations) or 1 ° (rotations) and therefore correspond to the resolution of our data sets. If the definition of FP measurements is changed and restricted to those observations with absolute values of 2 mm/degrees or more (for instance, the erroneous detection of a 2° rotation error when no error was introduced), the overall FP rate decreased from 16% to 2% (4/175). Further improvement of FN and FP rates would only be expected with higher‐resolution images. These figures compare well to the results of Fitzpatrick et al.^(26)^ and indicate the accuracy of the chosen landmarks within our validation protocol. This protocol could therefore contribute to the validation of alternative linear registration methods. The validation of nonlinear and elastic registration solutions is hampered by the clear definition of a large series of proper landmarks at images of various modalities and, if possible, will involve a time‐consuming procedure. However, elastic registration methods are required to handle elastic deformations that inevitably will occur.

Several remarks can be made with respect to the design of the test procedure for our validation protocol. First, we acknowledge the fact that in the majority of misregistrations only one error was introduced. This may be a simplification of reality where translation and rotation errors can interfere in one registration. However, all errors in the subseries of six misregistrations with “combined” (up to three) translation and/or rotation errors were detected. Furthermore, it should be emphasized that the main goal of the validation protocol was to detect “unacceptable” registration errors, and it does not replace the registration procedure itself. Considering the case of a registration solution with multiple errors, it is sufficient to detect the presence of one such error that exceeds a certain threshold (and discard the registration), rather than to quantify all possible registration errors. An accurate visual validation protocol should result in a low probability of an unjust approval of a registration solution containing one or multiple errors.

Second, the combination of a single experienced observer and a limited number of data sets may cause a training effect when the observer becomes familiar with the data sets and quickly detects possible errors. In this study, the observations were obtained in about 20 sessions with assessment of one or two registrations during each session. Moreover, to further reduce a possible observation bias, all sessions were mingled with daily activities over a period of about three months.

Third, the intentional misregistrations within the test procedure were obtained by randomly changing the transformation parameters of the registered CT data sets. Although resulting in a representative series of misregistrations, the introduction of mere translation and rotation errors may be a simplification of the “real world.” Higher‐order geometric distortions in MRI (f.i. skew, “S” and barrel shaping) may result in complexly shaped data sets that require a nonrigid (elastic) transformation for adequate image registration. One such method is described by Maguire et al.(3) However, the impact of geometrical distortion in MR on registration accuracy did not reach significance in the validation study by West et al.(17) In practice, this potential problem can partly be solved by developing QA procedures that not only pertain to the calibration of the diagnostic equipment (magnet shimming (automatically conducted at regular time intervals in our department), adjusted field gradients), but also include the definition of proper acquisition parameters (such as the use of small interslice gaps). Furthermore, the QA should include a standardized procedure on a regular basis to estimate registration accuracy. Water phantom measurements with CT and MR are suited for this purpose.

Finally, we mention the fact that the lack of a gold standard for registration implies that the original “optimal” registration outcomes that were used may contain registration errors themselves. The observed registration errors reflect “errors” in relation to the original registration outcome and are not necessarily equal to the absolute registration errors. This lack of a gold standard precludes a final conclusion regarding whether the NMI method is clinically “correct” in predicting the optimal registration solution. However, in view of the capability of the validation protocol to detect intentionally applied registration errors, it may be considered safe to conclude that the optimal registration solution, although by definition unknown, lies very close to the solution as predicted by the NMI method.

## V. CONCLUSION

We conclude that the NMI method, as estimated by the measurement of coordinate differences between a series of well‐defined landmarks in CT and MR data sets, results in excellent registration accuracy with standard errors close to voxel size. In addition, our visual validation protocol can be applied to individual registration solution outcomes and ensures the detection of residual rotation as well as translation errors with high sensitivity and specificity in a time‐efficient manner. This protocol may also be used for the validation of other linear registration methods. This security measure has made us confident enough to implement the NMI method in clinical practice for registration of images of the head.

## Supporting information

Supplementary Material Files
