# Supplementary material for: Clinical validation of the normalized mutual information method for registration of CT and MR images in radiotherapy of brain tumors
Source: J Appl Clin Med Phys. 2004 Oct 21;5(3):66–79. doi: 10.1120/jacmp.v5i3.1959 (PMC5723487; doi:10.1120/jacmp.v5i3.1959)
Supplement: Supplementary file 1 — Supplementary Material Files [file ACM2-5-66-s001.doc]

Abstract of oral presentation at the 6th Biennial ESTRO Meeting on Physics and Radiation Technology for Clinical Radiotherapy (Sevilla, Spain, September 2001)

**Clinical implementation of the normalized mutual information method for**

**matching CT and MRI images in radiotherapy**

T. Veninga^1^, H. Huisman^2^, R. W. M. van der Maazen^1^ and H. Huizenga^1^

University Medical Centre Nijmegen, Nijmegen, The Netherlands

*Introduction*: Image registration has the potential to integrate information of different imaging modalities in order to improve target volume determination in radiotherapy planning. This paper describes the application of “Normalized Mutual Information method”, a novel 3D fully automated registration procedure, at a radiotherapy department.

*Material and methods:* The normalized mutual information (NMI) method was used for image registration as this method seems to be the most robust one at present. The method was applied for 15 patients with various brain tumors, which received CT and MRI brain imaging before the start of radiotherapy. In addition to the automatic registration, several independent measures were employed to detect coordinate differences between CT and MRI datasets in order to validate the automatic registration. Secondly, a protocol was developed for validation of the quality of individual registrations. Thirdly, the sensitivity of the validation protocol was tested in a series of misregistration procedures with intentionally applied registration errors that might occur in clinical practice.

*Results:* The 3D automatic registration seems to be more accurate than can be detected with the protocol for manual validation. Differences between manual and automatic registration along the x- and y-axes were less than 1.1 mm. The mean difference along the z-axis were less than 0.6 mm (which is less than the applied slice thickness in scanning). Maximum differences in the xy-plane and along the z-axis were 2.5 mm and 3 mm, respectively. The validation protocol that was developed permitted detection of the majority of intentionally applied translation errors larger than 1 mm and rotational errors that exceeded 1 degree. In conclusion, application of the NMI method results in excellent registration accuracy for application in radiotherapy of the brain. A validation protocol, next to visual (qualitative) inspection of registration quality, ensures the quality of individual registrations by detecting registration errors that exceeded 1 mm of translation or 1 degree of rotation. The importance of this validation protocol is underlined by the fact that as yet no gold standard for registration exists. The NMI method is currently incorporated in daily routine within our institute.
